# Diagnostic Performance of Three rK39 Rapid Diagnostic Tests and Two Direct Agglutination Tests for the Diagnosis of Visceral Leishmaniasis in Southern Iran

**DOI:** 10.1155/2022/3569704

**Published:** 2022-04-11

**Authors:** Zahra Rezaei, Bahman Pourabbas, Vera Kühne, Parham Pourabbas, Philippe Büscher

**Affiliations:** ^1^Professor Alborzi Clinical Microbiology Research Center, Shiraz University of Medical Sciences, Shiraz, Iran; ^2^Department of Clinical Sciences, Institute of Tropical Medicine, Antwerp, Belgium; ^3^Department of Biomedical Sciences, Institute of Tropical Medicine, Antwerp, Belgium

## Abstract

To evaluate the diagnostic performance of five alternative serodiagnostic tests, serum samples from 100 confirmed visceral leishmaniasis (VL) patients, 197 healthy endemic individuals, and 58 non-VL patients living in southern Iran were compared. The VL patients were defined as individuals with a positive result of the immunofluorescent antibody test (IFAT), having clinical signs and symptoms and appropriate response to treatment. The index tests were two direct agglutination tests, DAT-ITM (Institute of Tropical Medicine, Antwerp, Belgium) and DAT-KIT (Royal Tropical Institute, Amsterdam, The Netherlands), and three rapid diagnostic tests (RDTs), Kalazar Detect (InBios International Inc., USA), IT Leish (Bio-Rad, catalog 710124), and Leishmania test (Cypress Diagnostic Company, Belgium). Sensitivities of DAT-ITM and DAT-KIT were low, respectively, 56% and 59%, while specificities were acceptable, respectively, 98% and 93%. Observed sensitivities and specificities of RDTs were higher (71%, 81%, 70% and 99%, 99%, 98% for Kalazar Detect, IT Leish, and Leishmania test, respectively). Even with a maximum sensitivity of 81%, RDTs missed almost one-fifth of VL patients that were positive in IFAT. We conclude that RDTs in VL patients do not possess adequate performance in southern Iran and require some improvement, but they can still be helpful in the diagnosis and screening of the disease in this region due to their high specificity and speed.

## 1. Introduction

Visceral leishmaniasis (VL), also known as kala-azar, is considered one of the most important parasitic neglected tropical diseases caused by the *Leishmania donovani* complex [1]. VL is endemic in about 80 countries, and the worldwide number of new cases is estimated at around 500,000 annually [2]. If left untreated, symptomatic VL results in death within two years [3]. In Iran, more than 90% of VL patients are under the age of five [4].

Early diagnosis of VL is critical for effective treatment of the patients [3, 5]. A definitive diagnosis of VL relies on demonstration of the parasite in splenic aspirates, which is highly invasive and carries a risk of hemorrhage [6, 7]. Although bone marrow and lymph node aspirates are invasive, they are not as sensitive as their splenic counterparts for the correct diagnosis of all patients [5] and that is why a less invasive test for treatment decision is a major priority for VL management [8]. High levels of antibodies are produced during acute disease, and VL diagnosis can be supported by detecting antibodies that specifically bind to *Leishmania* antigens [9–11]. The direct agglutination test (DAT), IFAT, and enzyme-linked immunosorbent assay (ELISA), used for VL serodiagnosis, reportedly exhibited a sensitivity of 70.5%, 80.3%, and 83.6%, respectively, and a specificity of 100%, 90.5%, and 90.5%, respectively, in Iran [12]. Limitations of such tests include the need for a relatively long incubation time and a well-equipped laboratory [13, 14]. Also, different brands of RDTs are commercially available. Most of them are based on immunochromatography with the recombinant K39 antigen (rK39), a protein composed of multiple 39 amino acid repeats derived from the kinesin-like gene of *Leishmania chagasi* (*infantum*) [15]. These rK39-RDTs are easy to perform, quick, affordable, noninvasive, and applicable in the field [16]. Previous studies showed variable sensitivity and specificity depending on the study region and brand of the RDTs [17, 18]. Only the Kalazar Detect (InBios International Inc., Seattle, USA) has been evaluated in southern Iran, but no studies have been conducted to evaluate other brands of rK39-RDTs. The present study aimed to compare the performance of two DAT tests and three rK39-RDTs for VL diagnosis in southern Iran.

## 2. Materials and Methods

### 2.1. Ethical Statement

This study received ethical approval from the Ethics Committee of the Shiraz University of Medical Sciences (SUMS), Shiraz, Iran (No. IR.SUMS.REC. 1396.S494). Patients' laboratory data were anonymized and deidentified prior to analysis.

### 2.2. Participants

A total of 355 sera, including 100 from VL patients, 197 from endemic healthy subjects, and 58 from non-VL patients, were included in this study.

VL patients were 3 months to 11 years young children, 52% male and 48% female, 98% with a history of fever ≥14 days, 86% with hepatosplenomegaly, and 89% with anemia. All the patients had been referred from endemic regions and admitted to Nemazi Hospital at Shiraz University of Medical Sciences. They were all positive in IFAT with titers of 128 (*n* = 24), 256 (*n* = 33), 512 (*n* = 24), and 1024 (*n* = 19). They were all cured and afebrile upon either antimonial or amphotericin B therapy. In this study, a VL patient was defined as one with prolonged fever, splenomegaly, hepatosplenomegaly, and anemia, a positive result in IFAT, and responsiveness to treatment. Endemic healthy controls were 1–16 years young children (55% male and 45% female) from endemic areas for VL in southern Iran. They had no clinical symptoms, no VL history, and negative IFAT results (titer ≤64). Non-VL patients, from nonendemic areas for VL in Iran, were 15–65 years old and suffered from other disease: toxoplasmosis (*n* = 10), malaria (*n* = 11), cutaneous leishmaniasis (*n* = 10), fascioliasis (*n* = 6), hydatidosis (*n* = 9), fever of unknown origin (*n* = 3), hymenolepiasis (*n* = 1), myocardial infarction (*n* = 1), purpura (*n* = 1), rheumatoid arthritis (*n* = 3), scleroderma (*n* = 1), systemic lupus erythematosus (*n* = 1), and toxocariasis (*n* = 1). This group was negative in IFAT (titer ≤64).

Informed consent was obtained from all adults or the parents or guardians of the children. 5 ml of venous blood was obtained from all individuals in plain tubes to prepare serum.

### 2.3. Serologic Diagnosis

IFAT was performed according to a previous study [19]. Briefly, in vitro cultured promastigotes of *L. infantum* strain (MCAN/IR/14/M14) were coated on IFAT microscope slides. Two-fold serial dilutions, from 1 : 64 to 1 : 1024, were prepared from all the sera, of which 10 *μ*l was dispensed on the reaction zones of IFAT slides. Upon 30 min incubation and subsequent washing, 5 *μ*l of 1 : 30 diluted antihuman globulin-FITC conjugate (Invitrogen, US) was placed on the reaction zone for 30 minutes. Afterwards, slides were washed and assessed utilizing fluorescence microscopy. The serum was considered positive when its titer was >64. Sera were tested with five index tests: three rK39 rapid tests and two DAT tests.

The rapid tests, Kalazar Detect (InBios International Inc., USA), IT Leish (Bio-Rad, catalog 710124), and VL Leishmania test (Cypress Diagnostic Company, Belgium), were performed according to the manufacturers' instructions.

DAT was performed according to the previous study, using freeze-dried antigen (fixed and stained promastigotes of *Leishmania donovani* strain 1-S) supplied by the Academic Medical Center, Laboratory for Clinical Parasitology, Amsterdam, Netherlands [14], and by the Institute of Tropical Medicine, Antwerp, Belgium (http://www.itg.be/files/docs/TTP/brochures/PDT_BR_0008_E_1.2.pdf).

### 2.4. Statistical Analysis

With 95% confidence intervals (CI), sensitivity and specificity were calculated using the online MedCalc software (https://www.medcalc.org/calc/diagnostic_test.php). The degree of agreement between RDTs and DATs was calculated as Kappa value with 95% CI, using SPSS version 18 (SPSS Inc., USA). Kappa values are interpreted according to Landis and Koch [20] and represented as follows: negligible (*k* = 0–0.20), weak (*k* = 0.21–0.40), moderate (*k* = 0.41–0.60), good (*k* = 0.61–0.80), and excellent agreement (*k* = 0.81–1). The McNemar test was performed to compare the sensitivity and specificity of the diagnostic tests. Bland–Altman (B&A) analysis was conducted to assess the agreement between serum titers obtained in DAT-KIT and DAT-ITM [21].

## 3. Results

The test results are given in detail in Supplementary Materials. According to VL patient definition, among the 100 VL patients, 59, 56, 71, 81, and 70 were positive in DAT-KIT, DAT-ITM, Kalazar Detect, IT Leish, and Leishmania test, respectively (Table 1). Combining DAT-KIT and DAT-ITM increases the sensitivity to 73%. A significant difference was observed between the sensitivity of Kalazar Detect and IT Leish (71% versus 81%; *p*=0.008) and the sensitivity of the Leishmania test and IT Leish (70% versus 81%; *p*=0.002).

**Figure 1:**
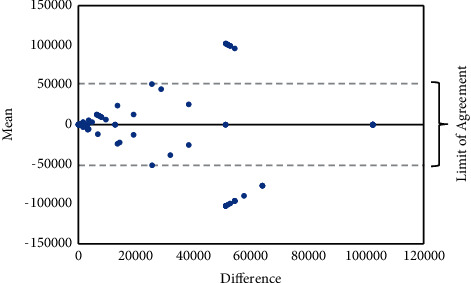
B&A graph of differences between titers in DAT-KIT and DAT-ITM versus the mean of the two titers. The solid line represents the mean difference and the two dotted lines represent the lower and upper limits of agreement.

Figure 1 shows the B&A graph that plots the differences in titers obtained in DAT-KIT and DAT-ITM against the mean of these titers. The graph shows that agreement between the titers in the two DAT tests is low for samples with a titer ≥51200 in one of the tests.

Among the two control group of participants, 245/249, 237/255, 250/252, 252/255, and 249/255 were negative by DAT-ITM, DAT-KIT, Kalazar Detect, IT Leish, and Leishmania test, respectively (Table 1). Of the 58 participants of the non-VL group, four with cutaneous leishmaniasis, two with malaria, and one with hydatidosis were positive by DAT-KIT, and one with toxoplasmosis was positive by DAT-ITM. One patient with malaria was positive by all three RDTs. Two patients with cutaneous leishmaniasis were positive by one or two RDTs. No significant difference was observed between the specificity of the RDTs (*p* > 0.05), and no significant difference was found on sensitivity after a combination of the three RDTs results compared to the individual RDTs (*p* > 0.05).

The agreement between these serological tests is given in Table 2. As already suggested in the B & A graph, an agreement between the two DAT tests is weak, while the agreement between DAT tests and RDTs ranged from moderate to good, while the agreement between RDTs was excellent.

## 4. Discussion

The present study aimed to compare the performance of two DAT tests and three rK39-RDTs for VL diagnosis in southern Iran.

DAT is the serological method of choice for VL diagnosis in several countries, including some parts of Iran. Although the DAT benefits from being a quantitative test, it has drawbacks that make it less convenient as a test in fields. The most consistent problems identified in laboratories can be attributed to the laboratory facilities, cold chain maintenance, requiring more pipetting, and several hours of incubation and staff mentality, which usually affect the final report titer of the DAT.

We found IT Leish, an rK39 antigen-based RDT, more sensitive (81%) than the two DATs and two RDTs performed in this study. DAT-ITM and DAT-KIT showed a sensitivity of 56% and 59%, respectively. A previous study, conducted by Sarkari and co-workers in the same region as the current study, showed a sensitivity of 70% for a DAT that was locally produced with *L. infantum* strain [22]. In another study conducted by Akhoundi et al., a sensitivity of 95.4% and specificity of 88.5% for DAT using *L. infantum* strain with 1 : 1600 as a cutoff titer were reported [23]. In DAT-ITM and DAT-KIT, promastigotes of *L. donovani* are used as antigen, while the causative agent of VL in Iran is mostly *L. infantum* and, to a lesser extent, *L. tropica* [24]. Therefore, if *L. infantum* or *L. tropica* instead of *L. donovani* was used to prepare DAT-ITM or DAT-KIT, its sensitivity could have been higher. Combining the results obtained in DAT-ITM and DAT-KIT increases the sensitivity to 73%, suggesting that sera reacted differently in the two DAT tests. Indeed, the B&A analysis showed an unexpected lack of agreement in serum titers obtained in the two DAT tests. Although both DAT tests are produced with the same strain of *L. donovani*, differences in growth media, promastigote fixation, staining, and test reagents may affect the reactivity of the DAT antigen with individual patients' serum. Twenty-seven patients who tested negative on DAT-KIT and DAT-ITM had the lowest antibody titer in IFAT. Sixteen of these twenty-seven patients had at least one positive result on RDT, probably due to the lower sensitivity of DAT.

Many non-VL and endemic healthy control group cases tested positive in DAT-ITM and DAT-KIT. Cross-reactions of *L. infantum* antigen with sera from persons with cutaneous leishmaniasis, malaria, and toxoplasmosis have been reported elsewhere [25]. On the other hand, some people living in VL endemic areas can carry asymptomatic infections and thus be seropositive for VL [26, 27].

The rK39 antigen, derived from a kinesin-like gene found in *Leishmania* species, is used in RDTs to detect a specific antibody against K39 antigen of *L. donovani* complex. The IT Leish was found to be more sensitive (81%) than Kalazar Detect (71%) and Leishmania test (70%) in the current study, consistent with observations by Chappuis et al. [28]. A previous global evaluation study has reported 87.2% and 92% sensitivity for IT Leish in East African countries and Brazil, respectively, while the sensitivity was 98.8% in the Indian subcontinent [29]. The sensitivity of IT Leish obtained in our study is close to that reported from African VL patients (87.2%) [29]. Different sensitivities of RDTs across the world have been attributed to differences in K39 sequence of the dominant parasites, host genetic background, and lower titer antibody responses against K39 in some patients [6, 30, 31].

As some researchers have suggested, the higher sensitivity of IT Leish could be explained by different formats of the tests [32]. To perform IT Leish, serum and conjugate are first mixed, and then, the dipstick is dipped in this mixture; however, for the Leishmania test and Kalazar Detect, the conjugate is incorporated in the immunochromatography strip, and only serum and buffer are applied on the strip. Generally, a more straightforward format with fewer steps or fewer materials was likely to be performed confidently. It was shown that cassette format RDTs generally act more reliably than dipstick formats [33]. Other factors that may reportedly affect the performance include the conjugate used, the concentration of the components, the type of sample (serum, plasma, or whole blood), and the volume of sample applied [34]. One out of the 58 non-VL patients with malaria was positive with three RDTs. Combining the results of each serum with three RDTs did not lead to a significant increase in overall diagnostic performance, and there were only two sera that were negative in IT Leish, while positive in the Leishmania test and/or Kalazar Detect.

## 5. Conclusions

Given the abovementioned, the RDT format is a factor that could influence test performance. Although IT Leish was more sensitive than DATs, Kalazar Detect, and the Leishmania test, it missed a significant VL cases, 19 (19%). Despite the limited sensitivity of RDTs in this study, their high specificity and speed made them helpful in diagnosing and screening disease in this region. However, further investigations to improve the diagnostic performance of RDTs in southern Iran are warranted.

## Figures and Tables

**Table 1:** Number of samples that tested positive and percent sensitivity and specificity of RDTs and DATs for visceral leishmaniasis.

Test	VL patients, *n* = 100	Endemic controls, *n* = 197	Non-VL patients, *n* = 58	Sensitivity, 95% CI	Specificity, 95% CI
DAT-KIT	59	11	7	59.0, 48.7–68.7	92.9, 89.1–95.8
DAT-ITM	56	3^*∗*^	1^*∗∗*^	56.0, 66.4–84.0	98.5, 96–99.6
Kalazar Detect	71	0	2	71.0, 61.1–79.6	99.2, 97.2–99.9
IT Leish	81	1	2	81.0, 71.9–88.2	98.82, 96.6–99.8
Leishmania test	70	4	2	70.0, 60.0–78.8	97.7, 95–99.1

CI, confidence interval; VL, visceral leishmaniasis. ^*∗*^196 tested; ^*∗∗*^53 tested.

**Table 2:** Kappa values expressing agreement between the serological tests.

Test	DAT-KIT	Kalazar Detect	IT Leish	Leishmania test
DAT-ITM	0.36	0.47	0.49	0.48
DAT-KIT		0.64	0.69	0.60
Kalazar Detect			0.84	0.87
IT Leish				0.84

## Data Availability

The data and material used to support the findings of this study are available from the corresponding author upon request.

## Supplementary Materials

Supplementary Materials Supplementary Material 1 contains the serological test results from VL patients. Supplementary Material 2 contains the serological test results from non-VL patients. Supplementary Material 3 contains the serological test results from endemic healthy controls.
