# Supplementary material for: Diagnostic Performance of Three rK39 Rapid Diagnostic Tests and Two Direct Agglutination Tests for the Diagnosis of Visceral Leishmaniasis in Southern Iran
Source: J Trop Med. 2022 Apr 11;2022:3569704. doi: 10.1155/2022/3569704 (PMC9017523; doi:10.1155/2022/3569704)
Supplement: Supplementary Materials — Supplementary Material 1 contains the serological test results from VL patients. Supplementary Material 2 contains the serological test results from non-VL patients. Supplementary Material 3 contains the serological test results from endemic healthy controls. [file 3569704.f1.zip › 3569704.f1/supplementary 2- Results of serological tests- Non VL patients.docx]

| **Results of serological tests- Non VL patients** |  | IFAT titer | DAT-KIT titer | DAT-ITM titer |  |  |  |
| --- | --- | --- | --- | --- | --- | --- | --- |
| No | Disease | cut-off ≥64 | cut-off ≥3200 | cut-off ≥3200 | IT-Leish | Leishmania Test | Kalazar Detect |
| 1 | Fascioliasis | <64 | 100 | 200 | NEG | NEG | NEG |
| 2 | Fascioliasis | <64 | 100 | 0 | NEG | NEG | NEG |
| 3 | Fascioliasis | <64 | 100 | 0 | NEG | NEG | NEG |
| 4 | Fascioliasis | <64 | 100 | 0 | NEG | NEG | NEG |
| 5 | Fascioliasis | <64 | 100 | 0 | NEG | NEG | NEG |
| 6 | Fascioliasis | <64 | 100 | 200 | NEG | NEG | NEG |
| 7 | Toxocariasis | <64 | 100 | 200 | NEG | NEG | NEG |
| 8 | FUO | <64 | 100 | ND | NEG | NEG | NEG |
| 9 | Scleroderma | <64 | 100 | 0 | NEG | NEG | NEG |
| 10 | Rheumatoid arthritis | <64 | 100 | ND | NEG | NEG | NEG |
| 11 | Rheumatoid arthritis | <64 | 100 | ND | NEG | NEG | NEG |
| 12 | Rheumatoid arthritis | <64 | 100 | 0 | NEG | NEG | NEG |
| 13 | Purpura | <64 | 100 | 0 | NEG | NEG | NEG |
| 14 | Myocardial Infarction | <64 | 100 | 0 | NEG | NEG | NEG |
| 15 | Hymenolepiasis | <64 | 100 | 0 | NEG | NEG | NEG |
| 16 | FUO | <64 | 100 | 0 | NEG | NEG | NEG |
| 17 | FUO | <64 | 100 | 0 | NEG | NEG | NEG |
| 18 | Cutaneous leishmaniasis | <64 | 100 | ND | NEG | NEG | NEG |
| 19 | Cutaneous leishmaniasis | <64 | 100 | 0 | NEG | NEG | NEG |
| 20 | Cutaneous leishmaniasis | <64 | 6400 | ND | NEG | NEG | NEG |
| 21 | Cutaneous leishmaniasis | <64 | 6400 | 0 | NEG | NEG | NEG |
| 22 | Cutaneous leishmaniasis | <64 | 100 | 0 | NEG | NEG | NEG |
| 23 | Cutaneous leishmaniasis | <64 | 3200 | 0 | POS | NEG | NEG |
| 24 | Cutaneous leishmaniasis | <64 | 100 | 0 | NEG | NEG | NEG |
| 25 | Cutaneous leishmaniasis | <64 | 100 | 0 | NEG | NEG | NEG |
| 26 | Cutaneous leishmaniasis | <64 | 6400 | 0 | NEG | POS | POS |
| 27 | Cutaneous leishmaniasis | <64 | 100 | 0 | NEG | NEG | NEG |
| 28 | Malaria (Plasmodium falciparum) | <64 | 3200 | 200 | NEG | NEG | NEG |
| 29 | Malaria (Plasmodium vivax) | <64 | 100 | 0 | NEG | NEG | NEG |
| 30 | Malaria (Plasmodium vivax) | <64 | 100 | 0 | NEG | NEG | NEG |
| 31 | Malaria (Plasmodium vivax) | <64 | 100 | 0 | NEG | NEG | NEG |
| 32 | Malaria (Plasmodium vivax) | <64 | 100 | 0 | NEG | NEG | NEG |
| 33 | Malaria (Plasmodium vivax) | <64 | 100 | 0 | NEG | NEG | NEG |
| 34 | Malaria (Plasmodium vivax) | <64 | 100 | 0 | NEG | NEG | NEG |
| 35 | Malaria (Plasmodium falciparum) | <64 | 100 | 0 | NEG | NEG | NEG |
| 36 | Malaria (Plasmodium falciparum) | <64 | 400 | 0 | POS | POS | POS |
| 37 | Malaria (Plasmodium vivax) | <64 | 100 | 200 | NEG | NEG | NEG |
| 38 | Malaria (Plasmodium vivax) | <64 | 100 | 0 | NEG | NEG | NEG |
| 39 | Toxoplasmosis | <64 | 100 | 0 | NEG | NEG | NEG |
| 40 | Toxoplasmosis | <64 | 100 | 0 | NEG | NEG | NEG |
| 41 | Toxoplasmosis | <64 | 100 | 3200 | NEG | NEG | NEG |
| 42 | Toxoplasmosis | <64 | 100 | 0 | NEG | NEG | NEG |
| 43 | Toxoplasmosis | <64 | 100 | 0 | NEG | NEG | NEG |
|  | Toxoplasmosis | <64 | 100 | 0 | NEG | NEG | NEG |
| 45 | Toxoplasmosis | <64 | 100 | 0 | NEG | NEG | NEG |
| 46 | Toxoplasmosis | <64 | 100 | 0 | NEG | NEG | NEG |
| 47 | Toxoplasmosis | <64 | 100 | 0 | NEG | NEG | NEG |
| 48 | Toxoplasmosis | <64 | 100 | 0 | NEG | NEG | NEG |
| 49 | Hydatidosis | <64 | 3200 | 0 | NEG | NEG | NEG |
| 50 | Hydatidosis | <64 | 100 | 0 | NEG | NEG | NEG |
| 51 | Hydatidosis | <64 | 100 | 0 | NEG | NEG | NEG |
| 52 | Hydatidosis | <64 | 100 | 0 | NEG | NEG | NEG |
| 53 | Hydatidosis | <64 | 100 | 0 | NEG | NEG | NEG |
| 54 | Hydatidosis | <64 | 100 | 0 | NEG | NEG | NEG |
| 55 | Hydatidosis | <64 | 100 | 0 | NEG | NEG | NEG |
| 56 | Hydatidosis | <64 | 100 | 0 | NEG | NEG | NEG |
| 57 | Hydatidosis | <64 | 100 | 0 | NEG | NEG | NEG |
| 58 | Systemic lupus erythematosus | <64 | 100 | 0 | NEG | NEG | NEG |
