# Supplementary material for: Diagnostic Performance of Three rK39 Rapid Diagnostic Tests and Two Direct Agglutination Tests for the Diagnosis of Visceral Leishmaniasis in Southern Iran
Source: J Trop Med. 2022 Apr 11;2022:3569704. doi: 10.1155/2022/3569704 (PMC9017523; doi:10.1155/2022/3569704)
Supplement: Supplementary Materials — Supplementary Material 1 contains the serological test results from VL patients. Supplementary Material 2 contains the serological test results from non-VL patients. Supplementary Material 3 contains the serological test results from endemic healthy controls. [file 3569704.f1.zip › 3569704.f1/Supplementary 3- Results of serological tests- Endemic healthy control.docx]

**Results of 197 serological tests from Endemic healthy control**

|  | IFAT titer | DAT-KIT titer | DAT-ITM titer |  |  |  |
| --- | --- | --- | --- | --- | --- | --- |
| Sample ID | cut-off ≥64 | cut-off ≥3200 | cut-off ≥3200 | IT-Leish | Leishmania Test | Kalazar Detect |
| 2 | <64 | 400 | 0 | NEG | NEG | NEG |
| 5 | <64 | 800 | 0 | NEG | NEG | NEG |
| 6 | <64 | 400 | 0 | NEG | NEG | NEG |
| 9 | <64 | 400 | 0 | NEG | NEG | NEG |
| 10 | <64 | 200 | 0 | NEG | NEG | NEG |
| 12 | <64 | 400 | 0 | NEG | NEG | NEG |
| 13 | <64 | 400 | 0 | NEG | NEG | NEG |
| 14 | <64 | 200 | 200 | NEG | NEG | NEG |
| 15 | <64 | 200 | 0 | NEG | NEG | NEG |
| 18 | <64 | 400 | 0 | NEG | NEG | NEG |
| 19 | <64 | 400 | 0 | NEG | NEG | NEG |
| 20 | <64 | 1600 | 0 | NEG | NEG | NEG |
| 21 | <64 | 0 | 0 | NEG | NEG | NEG |
| 22 | <64 | 800 | 0 | NEG | NEG | NEG |
| 24 | <64 | 800 | 0 | NEG | POS | NEG |
| 25 | <64 | 1600 | 0 | NEG | NEG | NEG |
| 26 | <64 | 1600 | 0 | NEG | NEG | NEG |
| 27 | <64 | 1600 | 0 | NEG | NEG | NEG |
| 31 | <64 | 1600 | 0 | NEG | NEG | NEG |
| 32 | <64 | 3200 | 1600 | NEG | NEG | NEG |
| 33 | <64 | 0 | 0 | NEG | NEG | NEG |
| 34 | <64 | 800 | 0 | NEG | NEG | NEG |
| 35 | <64 | 800 | 0 | NEG | NEG | NEG |
| 36 | <64 | 1600 | 0 | NEG | NEG | NEG |
| 37 | <64 | 1600 | 0 | NEG | NEG | NEG |
| 38 | <64 | 800 | 0 | NEG | NEG | NEG |
| 40 | <64 | 8800 | 0 | NEG | NEG | NEG |
| 42 | <64 | 800 | 0 | NEG | NEG | NEG |
| 44 | <64 | 800 | 0 | NEG | NEG | NEG |
| 50 | <64 | 200 | 0 | NEG | POS | NEG |
| 51 | <64 | 0 | 0 | NEG | NEG | NEG |
| 53 | <64 | 1600 | 0 | NEG | NEG | NEG |
| 55 | <64 | 800 | 0 | NEG | NEG | NEG |
| 57 | <64 | 200 | 0 | NEG | NEG | NEG |
| 58 | <64 | 800 | 0 | NEG | NEG | NEG |
| 60 | <64 | 800 | 0 | NEG | NEG | ND |
| 61 | <64 | 1600 | 0 | NEG | NEG | NEG |
| 64 | <64 | 400 | 0 | NEG | NEG | NEG |
| 66 | <64 | 800 | 0 | NEG | NEG | NEG |
| 70 | <64 | 1600 | 0 | NEG | NEG | NEG |
| 72 | <64 | 800 | 0 | NEG | NEG | NEG |
| 73 | <64 | 400 | 0 | NEG | NEG | NEG |
| 75 | <64 | 1600 | 0 | NEG | NEG | NEG |
| 76 | <64 | 800 | 0 | NEG | NEG | NEG |
| 77 | <64 | 800 | 0 | NEG | NEG | NEG |
| 78 | <64 | 1600 | 0 | NEG | NEG | NEG |
| 80 | <64 | 0 | 0 | NEG | NEG | NEG |
| 86 | <64 | 800 | 0 | NEG | NEG | NEG |
| 89 | <64 | 1600 | 0 | NEG | NEG | NEG |
| 89 | <64 | 800 | 0 | NEG | NEG | NEG |
| 92 | <64 | 400 | 0 | NEG | NEG | NEG |
| 96 | <64 | 800 | 0 | NEG | NEG | NEG |
| 97 | <64 | 1600 | 0 | NEG | NEG | NEG |
| 101 | <64 | 800 | 0 | NEG | NEG | NEG |
| 104 | <64 | 1600 | 0 | NEG | NEG | NEG |
| 107 | <64 | 800 | 0 | NEG | NEG | NEG |
| 109 | <64 | 800 | 0 | NEG | NEG | NEG |
| 111 | <64 | 800 | 0 | NEG | NEG | NEG |
| 112 | <64 | 800 | 0 | NEG | NEG | NEG |
| 113 | <64 | 400 | 0 | NEG | NEG | NEG |
| 114 | <64 | 400 | 0 | NEG | NEG | NEG |
| 115 | <64 | 1600 | 0 | NEG | POS | NEG |
| 116 | <64 | 400 | 0 | NEG | NEG | NEG |
| 117 | <64 | 1600 | 0 | NEG | NEG | NEG |
| 121 | <64 | 800 | 0 | NEG | NEG | NEG |
| 122 | <64 | 1600 | 0 | NEG | NEG | NEG |
| 123 | <64 | 400 | 0 | NEG | NEG | NEG |
| 124 | <64 | 800 | ND | NEG | NEG | ND |
| 125 | <64 | 0 | 0 | NEG | NEG | NEG |
| 125 | <64 | 1600 | 0 | NEG | NEG | NEG |
| 128 | <64 | 400 | 0 | NEG | NEG | NEG |
| 130 | <64 | 800 | 0 | NEG | NEG | NEG |
| 133 | <64 | 1600 | 200 | NEG | NEG | NEG |
| 135 | <64 | 800 | 0 | NEG | NEG | NEG |
| 137 | <64 | 800 | 0 | NEG | NEG | NEG |
| 138 | <64 | 800 | 0 | NEG | NEG | NEG |
| 139 | <64 | 1600 | 0 | NEG | NEG | NEG |
| 140 | <64 | 800 | 0 | NEG | NEG | NEG |
| 141 | <64 | 800 | 0 | NEG | NEG | NEG |
| 142 | <64 | 1600 | 0 | NEG | NEG | NEG |
| 143 | <64 | 400 | 0 | NEG | NEG | NEG |
| 144 | <64 | 800 | 0 | NEG | NEG | NEG |
| 145 | <64 | 1600 | 0 | NEG | NEG | NEG |
| 149 | <64 | 800 | 0 | NEG | NEG | NEG |
| 154 | <64 | 400 | 0 | NEG | NEG | NEG |
| 155 | <64 | 1600 | 0 | NEG | NEG | NEG |
| 156 | <64 | 400 | 0 | NEG | NEG | NEG |
| 159 | <64 | 800 | 0 | NEG | NEG | NEG |
| 164 | <64 | 0 | 0 | NEG | NEG | ND |
| 165 | <64 | 1600 | 0 | NEG | NEG | NEG |
| 167 | <64 | 800 | 0 | NEG | NEG | NEG |
| 169 | <64 | 400 | 0 | NEG | NEG | NEG |
| 170 | <64 | 1600 | 0 | NEG | NEG | NEG |
| 171 | <64 | 1600 | 0 | NEG | NEG | NEG |
| 173 | <64 | 0 | 0 | NEG | NEG | NEG |
| 175 | <64 | 800 | 0 | NEG | NEG | NEG |
| 176 | <64 | 800 | 0 | NEG | NEG | NEG |
| 179 | <64 | 800 | 0 | NEG | NEG | NEG |
| 182 | <64 | 800 | 0 | NEG | NEG | NEG |
| 183 | <64 | 400 | 0 | NEG | NEG | NEG |
| 185 | <64 | 800 | 0 | NEG | NEG | NEG |
| 186 | <64 | 800 | 0 | NEG | NEG | NEG |
| 188 | <64 | 400 | 0 | NEG | NEG | NEG |
| 192 | <64 | 1600 | 0 | NEG | NEG | NEG |
| 197 | <64 | 800 | 0 | NEG | NEG | NEG |
| 200 | <64 | 800 | 0 | NEG | NEG | NEG |
| 215 | <64 | 1600 | 0 | NEG | NEG | NEG |
| 228 | <64 | 0 | 0 | NEG | NEG | NEG |
| 235 | <64 | 800 | 0 | NEG | NEG | NEG |
| 282 | <64 | 1600 | 0 | NEG | NEG | NEG |
| 292 | <64 | 800 | 0 | NEG | NEG | NEG |
| 401 | <64 | 1600 | 0 | NEG | NEG | NEG |
| 402 | <64 | 800 | 0 | NEG | NEG | NEG |
| 404 | <64 | 400 | 0 | NEG | NEG | NEG |
| 405 | <64 | 1600 | 0 | NEG | NEG | NEG |
| 405 | <64 | 800 | 0 | NEG | NEG | NEG |
| 406 | <64 | 800 | 0 | NEG | NEG | NEG |
| 407 | <64 | 800 | 0 | NEG | NEG | NEG |
| 408 | <64 | 3200 | 0 | POS | NEG | NEG |
| 409 | <64 | 100 | 0 | NEG | NEG | NEG |
| 410 | <64 | 1600 | 0 | NEG | NEG | NEG |
| 414 | <64 | 3200 | 0 | NEG | NEG | NEG |
| 416 | <64 | 1600 | 0 | NEG | NEG | NEG |
| 418 | <64 | 1600 | 0 | NEG | NEG | NEG |
| 419 | <64 | 0 | 0 | NEG | NEG | NEG |
| 420 | <64 | 1600 | 0 | NEG | NEG | NEG |
| 421 | <64 | 800 | 0 | NEG | NEG | NEG |
| 422 | <64 | 1600 | 0 | NEG | NEG | NEG |
| 423 | <64 | 1600 | 0 | NEG | NEG | NEG |
| 424 | <64 | 1600 | 0 | NEG | NEG | NEG |
| 425 | <64 | 3200 | 0 | NEG | NEG | NEG |
| 426 | <64 | 800 | 0 | NEG | NEG | NEG |
| 427 | <64 | 800 | 0 | NEG | NEG | NEG |
| 428 | <64 | 800 | 0 | NEG | NEG | NEG |
| 430 | <64 | 1600 | 0 | NEG | NEG | NEG |
| 432 | <64 | 800 | 0 | NEG | NEG | NEG |
| 432 | <64 | 1600 | 0 | NEG | NEG | NEG |
| 434 | <64 | 1600 | 0 | NEG | NEG | NEG |
| 437 | <64 | 0 | 0 | NEG | NEG | NEG |
| 438 | <64 | 1600 | 0 | NEG | NEG | NEG |
| 439 | <64 | 3200 | 0 | NEG | NEG | NEG |
| 440 | <64 | 3200 | 3200 | NEG | NEG | NEG |
| 441 | <64 | 1600 | 0 | NEG | NEG | NEG |
| 442 | <64 | 800 | 0 | NEG | NEG | NEG |
| 443 | <64 | 1600 | 0 | NEG | NEG | NEG |
| 444 | <64 | 1600 | 0 | NEG | NEG | NEG |
| 445 | <64 | 1600 | 0 | NEG | NEG | NEG |
| 447 | <64 | 1600 | 0 | NEG | NEG | NEG |
| 448 | <64 | 1600 | 0 | NEG | NEG | NEG |
| 451 | <64 | 0 | 0 | NEG | NEG | NEG |
| 452 | <64 | 1600 | 0 | NEG | NEG | NEG |
| 453 | <64 | 1600 | 0 | NEG | NEG | NEG |
| 455 | <64 | 3200 | 0 | NEG | NEG | NEG |
| 456 | <64 | 100 | 0 | NEG | NEG | NEG |
| 457 | <64 | 0 | 0 | NEG | NEG | NEG |
| 458 | <64 | 1600 | 0 | NEG | NEG | NEG |
| 459 | <64 | 800 | 0 | NEG | NEG | NEG |
| 462 | <64 | 1600 | 0 | NEG | NEG | NEG |
| 464 | <64 | 800 | 0 | NEG | NEG | NEG |
| 466 | <64 | 1600 | 0 | NEG | NEG | NEG |
| 468 | <64 | 3200 | 0 | NEG | NEG | NEG |
| 470 | <64 | 800 | 0 | NEG | NEG | NEG |
| 472 | <64 | 1600 | 0 | NEG | NEG | NEG |
| 474 | <64 | 800 | 0 | NEG | POS | NEG |
| 505 | <64 | 800 | 0 | NEG | NEG | NEG |
| 506 | <64 | 1600 | 0 | NEG | NEG | NEG |
| 507 | <64 | 400 | 0 | NEG | NEG | NEG |
| 508 | <64 | 800 | 0 | NEG | NEG | NEG |
| 509 | <64 | 800 | 0 | NEG | NEG | NEG |
| 510 | <64 | 800 | 0 | NEG | NEG | NEG |
| 511 | <64 | 800 | 0 | NEG | NEG | NEG |
| 512 | <64 | 0 | 0 | NEG | NEG | NEG |
| 514 | <64 | 0 | 0 | NEG | NEG | NEG |
| 516 | <64 | 800 | 0 | NEG | NEG | NEG |
| 517 | <64 | 400 | 0 | NEG | NEG | NEG |
| 518 | <64 | 400 | 0 | NEG | NEG | NEG |
| 519 | <64 | 400 | 0 | NEG | NEG | NEG |
| 520 | <64 | 400 | 0 | NEG | NEG | NEG |
| 522 | <64 | 800 | 0 | NEG | NEG | NEG |
| 523 | <64 | 800 | 0 | NEG | NEG | NEG |
| 528 | <64 | 800 | 0 | NEG | NEG | NEG |
| 534 | <64 | 800 | 0 | NEG | NEG | NEG |
| 535 | <64 | 3200 | 0 | NEG | NEG | NEG |
| 537 | <64 | 1600 | 0 | NEG | NEG | NEG |
| 538 | <64 | 400 | 0 | NEG | NEG | NEG |
| 539 | <64 | 800 | 0 | NEG | NEG | NEG |
| 540 | <64 | 1600 | 0 | NEG | NEG | NEG |
| 541 | <64 | 3200 | 3200 | NEG | NEG | NEG |
| 544 | <64 | 800 | 0 | NEG | NEG | NEG |
| 544 | <64 | 400 | 0 | NEG | NEG | NEG |
| 546 | <64 | 1600 | 0 | NEG | NEG | NEG |
| 547 | <64 | 1600 | 0 | NEG | NEG | NEG |
| 548 | <64 | 800 | 0 | NEG | NEG | NEG |
| 549 | <64 | 800 | 0 | NEG | NEG | NEG |
| 550 | <64 | 800 | 0 | NEG | NEG | NEG |
| 552 | <64 | 1600 | 0 | NEG | NEG | NEG |
| 553 | <64 | 800 | 0 | NEG | NEG | NEG |
