# Supplementary material for: Diagnostic Performance of Three rK39 Rapid Diagnostic Tests and Two Direct Agglutination Tests for the Diagnosis of Visceral Leishmaniasis in Southern Iran
Source: J Trop Med. 2022 Apr 11;2022:3569704. doi: 10.1155/2022/3569704 (PMC9017523; doi:10.1155/2022/3569704)
Supplement: Supplementary Materials — Supplementary Material 1 contains the serological test results from VL patients. Supplementary Material 2 contains the serological test results from non-VL patients. Supplementary Material 3 contains the serological test results from endemic healthy controls. [file 3569704.f1.zip › 3569704.f1/Supplementay 1- Results of serological tests- VL patients.docx]

**Results of serological tests- VL patients**

|  | IFAT titer | DAT-KIT titer | DAT-ITM titer |  |  |  |
| --- | --- | --- | --- | --- | --- | --- |
| No | cut-off ≥64 | cut-off ≥3200 | cut-off ≥3200 | IT-Leish | Leishmania Test | Kalazar Detect |
| 1 | 1024 | 102400 | 1600 | POS | POS | POS |
| 2 | 512 | 12800 | 1600 | POS | POS | POS |
| 3 | 512 | 102400 | 400 | POS | POS | POS |
| 4 | 512 | 800 | 200 | POS | POS | POS |
| 5 | 256 | 12800 | 1600 | POS | POS | POS |
| 6 | 256 | 400 | 0 | POS | POS | POS |
| 7 | 128 | 200 | 0 | NEG | NEG | NEG |
| 8 | 128 | 102400 | 100 | POS | POS | POS |
| 9 | 512 | 102400 | 102400 | POS | POS | POS |
| 10 | 128 | 200 | 200 | POS | NEG | NEG |
| 11 | 128 | 100 | 102400 | POS | NEG | NEG |
| 12 | 256 | 100 | 6400 | NEG | NEG | NEG |
| 13 | 256 | 100 | 51200 | POS | POS | POS |
| 14 | 1024 | 102400 | 200 | POS | POS | POS |
| 15 | 1024 | 100 | 200 | POS | POS | NEG |
| 16 | 256 | 102400 | 1600 | POS | POS | POS |
| 17 | 256 | 6400 | 102400 | POS | POS | POS |
| 18 | 512 | 51200 | 0 | POS | POS | POS |
| 19 | 256 | 51200 | 6400 | POS | POS | POS |
| 20 | 512 | 102400 | 102400 | POS | POS | POS |
| 21 | 512 | 800 | 12800 | POS | POS | POS |
| 22 | 512 | 102400 | 800 | POS | POS | POS |
| 23 | 128 | 200 | 0 | POS | NEG | NEG |
| 24 | 512 | 3200 | 3200 | POS | POS | NEG |
| 25 | 512 | 102400 | 1600 | POS | POS | POS |
| 26 | 256 | 12800 | 12800 | POS | POS | POS |
| 27 | 128 | 6400 | 102400 | POS | POS | POS |
| 28 | 256 | 25600 | 102400 | POS | POS | POS |
| 29 | 512 | 800 | 6400 | POS | POS | POS |
| 30 | 256 | 400 | 200 | NEG | NEG | NEG |
| 31 | 128 | 3200 | 3200 | POS | NEG | NEG |
| 32 | 128 | 100 | 800 | NEG | NEG | NEG |
| 33 | 128 | 102400 | 102400 | POS | NEG | NEG |
| 34 | 256 | 100 | 800 | NEG | POS | POS |
| 35 | 512 | 25600 | 102400 | POS | POS | POS |
| 36 | 1024 | 102400 | 102400 | POS | POS | POS |
| 37 | 1024 | 51200 | 25600 | POS | POS | POS |
| 38 | 512 | 51200 | 51200 | POS | POS | POS |
| 39 | 1024 | 12800 | 3200 | POS | NEG | POS |
| 40 | 1024 | 102400 | 102400 | POS | POS | POS |
| 41 | 512 | 12800 | 800 | POS | POS | POS |
| 42 | 256 | 100 | 0 | NEG | NEG | NEG |
| 43 | 128 | 200 | 400 | POS | POS | POS |
| 44 | 128 | 100 | 0 | NEG | NEG | NEG |
| 45 | 128 | 400 | 100 | NEG | NEG | NEG |
| 46 | 256 | 25600 | 102400 | POS | POS | POS |
| 47 | 256 | 1600 | 400 | POS | POS | POS |
| 48 | 1024 | 51200 | 51200 | POS | POS | POS |
| 49 | 256 | 400 | 100 | POS | POS | POS |
| 50 | 128 | 100 | 100 | NEG | NEG | NEG |
| 51 | 256 | 102400 | 102400 | POS | NEG | POS |
| 52 | 128 | 100 | 100 | NEG | NEG | POS |
| 53 | 128 | 3200 | 100 | POS | POS | POS |
| 54 | 128 | 25600 | 12800 | POS | POS | POS |
| 55 | 128 | 200 | 800 | POS | NEG | NEG |
| 56 | 128 | 12800 | 51200 | POS | POS | POS |
| 57 | 256 | 100 | 0 | NEG | NEG | NEG |
| 58 | 256 | 100 | 100 | NEG | NEG | NEG |
| 59 | 128 | 100 | 102400 | NEG | NEG | NEG |
| 60 | 128 | 100 | 3200 | NEG | NEG | NEG |
| 61 | 128 | 100 | 800 | POS | POS | NEG |
| 62 | 128 | 800 | 400 | POS | NEG | POS |
| 63 | 512 | 102400 | 102400 | POS | POS | POS |
| 64 | 256 | 102400 | 102400 | POS | POS | POS |
| 65 | 1024 | 102400 | 102400 | POS | POS | POS |
| 66 | 256 | 12800 | 12800 | POS | POS | POS |
| 67 | 256 | 200 | 200 | POS | POS | NEG |
| 68 | 256 | 102400 | 102400 | POS | POS | POS |
| 69 | 1024 | 102400 | 102400 | POS | POS | POS |
| 70 | 256 | 800 | 3200 | POS | POS | POS |
| 71 | 1024 | 12800 | 0 | POS | NEG | POS |
| 72 | 128 | 1600 | 25600 | POS | POS | POS |
| 73 | 256 | 1600 | 102400 | POS | POS | POS |
| 74 | 1024 | 102400 | 3200 | POS | POS | POS |
| 75 | 256 | 100 | 102400 | POS | POS | POS |
| 76 | 512 | 25600 | 1600 | POS | POS | POS |
| 77 | 512 | 12800 | 25600 | POS | POS | POS |
| 78 | 512 | 6400 | 102400 | POS | POS | POS |
| 79 | 512 | 102400 | 6400 | POS | POS | POS |
| 80 | 1024 | 3200 | 102400 | POS | NEG | NEG |
| 81 | 256 | 6400 | 800 | POS | POS | POS |
| 82 | 512 | 12800 | 3200 | POS | POS | POS |
| 83 | 256 | 12800 | 12800 | POS | POS | POS |
| 84 | 512 | 3200 | 25600 | POS | POS | POS |
| 85 | 256 | 1600 | 800 | POS | POS | POS |
| 86 | 512 | 1600 | 400 | POS | POS | POS |
| 87 | 1024 | 102400 | 3200 | POS | POS | POS |
| 88 | 1024 | 100 | 102400 | NEG | NEG | NEG |
| 89 | 256 | 100 | 102400 | NEG | NEG | NEG |
| 90 | 256 | 6400 | 3200 | POS | POS | POS |
| 91 | 256 | 100 | 400 | NEG | NEG | NEG |
| 92 | 1024 | 800 | 100 | NEG | NEG | NEG |
| 93 | 1024 | 12800 | 100 | POS | POS | POS |
| 94 | 256 | 25600 | 51200 | POS | POS | POS |
| 95 | 512 | 51200 | 51200 | POS | POS | POS |
| 96 | 1024 | 6400 | 102400 | POS | POS | POS |
| 97 | 512 | 400 | 6400 | POS | POS | POS |
| 98 | 128 | 12800 | 6400 | POS | POS | POS |
| 99 | 1024 | 12800 | 102400 | POS | NEG | NEG |
| 100 | 256 | 3200 | 200 | NEG | NEG | NEG |
